# *Porphyromonas gingivalis*: keeping the pathos out of the biont

**DOI:** 10.3402/jom.v5i0.19804

**Published:** 2013-04-03

**Authors:** Carla Cugini, Vanja Klepac-Ceraj, Elze Rackaityte, James E. Riggs, Mary E. Davey

**Affiliations:** 1 Department of Microbiology, The Forsyth Institute, Cambridge, MA, USA; 2 Department of Oral Medicine Infection and Immunity, Harvard School of Dental Medicine, Boston, MA, USA; 3 Department of Biological Sciences, Wellesley College, Wellesley, MA, USA; 4 Department of Biology, Rider University, Lawrenceville, NJ, USA; 5 Department of Oral Biology, College of Dentistry, University of Florida, Gainesville, FL, USA

**Keywords:** pathobiont, *P. gingivalis*, biofilm, tumor, immunoediting hypothesis

## Abstract

The primary goal of the human microbiome initiative has been to increase our understanding of the structure and function of our indigenous microbiota and their effects on human health and predisposition to disease. Because of its clinical importance and accessibility for *in vivo* study, the oral biofilm is one of the best-understood microbial communities associated with the human body. Studies have shown that there is a succession of select microbial interactions that directs the maturation of a defined community structure, generating the formation of dental plaque. Although the initiating factors that lead to disease development are not clearly defined, in many individuals there is a fundamental shift from a health-associated biofilm community to one that is pathogenic in nature and a central player in the pathogenic potential of this community is the presence of *Porphyromonas gingivali*s. This anaerobic bacterium is a natural member of the oral microbiome, yet it can become highly destructive (termed pathobiont) and proliferate to high cell numbers in periodontal lesions, which is attributed to its arsenal of specialized virulence factors. Hence, this organism is regarded as a primary etiologic agent of periodontal disease progression. In this review, we summarize some of the latest information regarding what is known about its role in periodontitis, including pathogenic potential as well as ecological and nutritional parameters that may shift this commensal to a virulent state. We also discuss parallels between the development of pathogenic biofilms and the human cellular communities that lead to cancer, specifically we frame our viewpoint in the context of ‘wounds that fail to heal’.

Despite great advances in our knowledge regarding the causes and risk factors associated with periodontal diseases, there are no signs of a decline in its prevalence. In fact, longer retention of teeth, coupled with an aging population, may give rise to a future increase in the number of people affected. Research is just now discovering that changes in the microbiota of the oral cavity are indicators of systemic diseases, such as diabetes and cardiovascular disease [see review (1)]. Moreover, there are data indicating that chronic inflammation in the oral cavity can exacerbate these diseases. Hence, although we typically think of diseases of the oral cavity as non-life-threatening, one can argue that we are being short sighted; the oral cavity is indeed connected to the rest of body and oral health is tightly linked to systemic health.

Historically, the etiology of periodontal disease has evaded consistent classification. At the turn of the 20th century, the prevailing idea was that bacteria were non-specific agents acting only second to poor patient hygiene (2). During the four decades to follow, investigators hypothesized that plaque from patient to patient was composed of similar microorganisms and therefore could not be of a sole etiological agent. It was not until late 1970s that differences in microbial composition between healthy and periodontitis-afflicted subjects were established, indicating that the development of periodontal diseases and the changes in microbial community composition in the subgingival periodontal pocket are highly intertwined [reviewed in (3)]. It is now well-accepted that this disease progression is elicited by bacteria and results from a fundamental disturbance in microbial interactions and thereby the interplay of this complex microbial community with its host.

With hundreds of bacterial species persisting in the oral cavity, the potential for interactions within the oral biofilm is innumerable (4, 5). Periodontitis etiology only makes sense when viewed in light of this complex community and is best thought of as an inflammatory disease caused by development of a pathogenic community that grows together, evolves together, and becomes destructive together. Such a pathogenic community is more than just the sum of its members and persists because of its diversity (6). It is clear that complex bacterial communities co-exist in healthy individuals; yet the environmental, evolutionary, or host-associated triggers that force the community to become pathogenic are not fully understood. The primary microbial factor contributing to periodontal disease progression is a shift from a benign commensal biofilm to a community with higher levels of virulent bacteria, and the primary immunological factor is the destructive host inflammatory response (7). However, the contribution of bacteria to disease progression remains poorly understood as it involves such a complex interplay between the host's immune system and the resident microbiota (8). Another complicating factor is that the clinical manifestations of periodontitis is highly variable and current definitions of periodontitis are not sufficient to describe such variation (9, 10). What can be agreed upon is that there is a fundamental shift of the microbial community that is correlated to progressive disease intensity.

## *Porphyromonas gingivalis* virulence factors and its potential for causing dysbiosis

The consortium of bacteria within the oral cavity of healthy and diseased hosts has been well-characterized (see reviews 4, 21). In general, the assemblage in the supragingival plaque in healthy adults is composed primarily of Gram-positive species belonging to the genera of *Actinomyces* and *Streptococcus*, and to the Gram-negative genus *Veillonella* (11, 12), and patients with periodontal disease have a greater proportion of Gram-negative, proteolytic, bacteria such as *P. gingivalis* or *Tanneralla forsythia*, as well as species of *Prevotella*, *Fusobacterium*, and *Treponema* in their subgingival plaque (3, 11–16). Supragingival plaque has been implicated as a host environment for persistence of the Gram-negative bacteria, as low levels of these organisms are found in healthy adults (11, 15, 17–19). While these survey studies have been instrumental in the identification of key anaerobes contributing to the progression of disease, the question of what are the triggers that induce a change from oral health to disease remains unexplained. In fact, recent studies have actually challenged the paradigm that patients with periodontitis are primarily colonized by Gram-negative species (20–23).

**Fig. 1:**
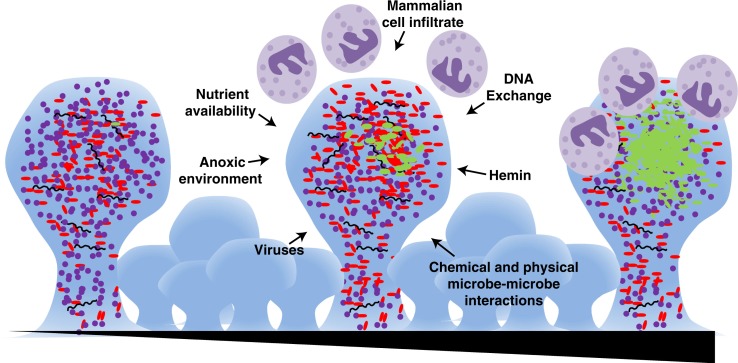
*P. gingivalis*-mediated periodontitis is a biofilm associated disease of slow progression. Early within the oral cavity there is a mixed community of bacteria with an abundance of Gram-positive organisms (purple cocci). Over time (represented by the triangle at bottom), there is a maturation of the biofilm. External and internal pressures such as an anoxic environment, mammalian cell infiltrate, viral and phage activity, DNA exchange, nutrient availability, and physical and chemical interactions of the microbes, drive development. A currently unidentified event occurs that allows for the out-growth of Gram-negative organisms (red and green rods), in particular *P. gingivalis* (shown in green). There is also active recruitment of cells of the immune system, such as neutrophils (shown in purple) to the site, which then brings in a battery of host-derived signaling molecules, impacting the microbial community.

*P. gingivalis* is best described as a pathobiont, i.e. a natural member of the human microbiota that under certain perturbations to the host and/or microflora can cause pathology (20–23). While it is thought that most people harbor *P. gingivalis* at some level in the mouth, a shift in the microbial composition is associated with its out-growth and development of periodontitis (Fig. 1) (24–28). *P. gingivalis* is known to be highly proteolytic, with primary activity from a class of endopeptidases known as gingipains that are critical virulence factors important for nutrient acquisition, located on the cell surface, in the outer membrane, and importantly extracellularly and non-cell associated (29–36). Proteolysis actively changes the environmental conditions; importantly, causing a pH increase, thus creating an environment more hospitable to many Gram-negative anaerobes. As cell numbers increase, the biofilm at the core becomes anoxic and nutrients are a commodity, which in turn makes the action of the proteases even more important. These proteases have also been shown to impact the host, both by the tissue destruction that facilitates nutrient liberation and on the immune system by the break-down of the cytokine response (37, 38). Complexed with lipopolysaccharides (LPS) and adhesins, gingipains have been shown to temper host immunity concurrent with host tissue destruction (39).

It was recently proposed that *P. gingivalis* is a keystone microorganism, such that within a healthy microflora it can cause dysbiosis and later disease (40). It was also recently shown that *P. gingivalis*, at very low colonization levels, can be sufficient to modify the oral commensal microbial community composition leading to inflammatory periodontal bone loss, however on its own, it cannot induce disease in germ free mice (41). In contrast, several studies have demonstrated that *P. gingivalis* can be present in the subgingival biofilm and colonize the epithelium in the absence of periodontal disease in an otherwise healthy mouth (42–45). These data indicate that the pathogenic potential of *P. gingivalis* is not solely dependent on its ability to colonize and proliferate; its pathogenicity is clearly related to both its physiological state and its association with the microbial consortium.

Interestingly, *P. gingivalis* presence is also associated with a shift to a microbial community consisting of more anaerobic bacteria and an overall increase in microbial load (46). Along the same lines, a recent metatranscriptome analysis showed that *P. gingivalis* introduced into a healthy polymicrobial model biofilm community increases the transcription of protein-coding genes for growth and cell-division (47). Furthermore, the addition of *P. gingivalis* caused the model microbial community to upregulate genes involved in nitrogen metabolism. This is not surprising as *P. gingivalis* is known for its proteolytic capacity to liberate amino acids from proteins, which can, in turn, be used by other microbial community members as well as itself (29–36). Interestingly, organisms associated with oral health such as *Streptococcus gordonii* are potential entry points for *P. gingivalis* to the biofilm and *Fusobacterium nucleatum* biofilms are enhanced with the addition of *P. gingivalis* (48, 49). Furthermore, these three organisms are found to up or down regulate a number of metabolic pathways upon contact with each other, indicating that they are actively responding to the presence of each other in the biofilm (50, 51). Findings from these studies suggest that bacterial cell–cell interactions with *P. gingivalis* may be a key to either maintaining health or progression to disease. These studies suggest that interactions with *P. gingivalis* may be key to the progression of periodontal disease, and that *P. gingivalis* plays a crucial role in causing dysbiosis within the oral microbial community, perhaps by serving as a communication disrupter between the polymicrobial biofilm and the host immune system; however, given the complexity of the interaction of the oral biofilm with the host, the precise role of *P. gingivalis* in development and/or progression of periodontal disease remains unclear.

## Importance of iron acquisition mechanisms for *P. gingivalis* persistence

Within the oral cavity nutrient availability is in constant flux, and like any well-developed pathogen *P. gingivalis* has devised mechanisms to resist this change. As observed with chemostat studies, *P. gingivalis* is able to control the expression of several virulence factors to survive in nutrient limiting environments (52). The acquisition of iron, for both humans and microbes, is a major signal to alter gene expression and protein production; in bacteria iron acquisition mechanisms are specifically associated with modulating expression of virulence determinants (53). When iron is limited, drastic changes in salivary bacterial profile result, such that there is an increase in *Streptococcus*, *Gemella* spp., and *Granulicatella* spp., which can, in turn, alter the landscape of the oral biofilm. If *P. gingivalis* is a resident within this community, it will then, in turn, alter its gene expression in response to the proliferation of Gram-positives (54). Furthermore, it has been shown that when *P. gingivalis* is grown in a biofilm with *T. forsythia*, there are distinct changes in the proteome, particularly for processes related to iron acquisition (55). Exhaustive research continues to be conducted on *P. gingivalis* strategies and molecular mechanisms for iron acquisition (56, 57). Hemin, or iron protoporphyrin IX, is the main form of iron used by *P. gingivalis*, which is acquired from hemoglobin through the enzymatic activity of the Arg- and Lys-gingipains. Lactoferrin is a host-derived iron-binding glycoprotein, found in salivary and gingival crevicular fluid that is able to chelate iron and plays an important role in innate immunity. Studies have shown that lactoferrin is able to inhibit bacterial adhesion to surfaces by discouraging the formation of biofilms in *Streptococcus mutans* (58). Additionally, lactoferrin displays proteinase-inhibiting activity against *P. gingivalis* Arg- and Lys-specific proteinases and is able to efficiently disrupt *P. gingivalis* biofilms (59).

## Effect of host-nutrition on oral microbiota

There are numerous factors that affect the health of the host immune system such as nutrition, lifestyle, and genetics, and interestingly these factors also impact periodontal health. Host-nutrition is especially important to consider when assessing diseases associated with the oral microbial community; bacteria inhabiting the oral cavity must adapt to the ever-changing nutrient availability. A neolithic diet and hygiene showed an intra-individual shift of bacterial composition of *F. nucleatum, A. naeslundii, Veillonella* spp., and *Streptococcus* spp., suggesting that diet free of refined sugars plays an important role in the composition of microbial microflora (60). A cross-sectional study found that calcium from milk, total dairy calcium, and fermented foods were inversely associated with periodontal disease after adjustment for population, hygiene, and other nutritional factors (61). Furthermore, the effect of dietary antioxidants on periodontal disease may play an important role in prevention of periodontal diseases, most likely acting by strengthening the immune system (62). Green tea-derived polyphenols, catechins, are able to inhibit the production of chemokine ligands associated with virulence in *P. gingivalis* (63, 64). Additionally, an epidemiological study of 940 Japanese men showed an inverse relationship between green tea uptake and periodontal disease (65). Polyphenols derived from cranberries show an effect on adherence and biofilm formation of *P. gingivalis* and *F. nucleatum* (66, 67). Resveratrol, a polyphenol found in wine, increased the viability of human periodontal ligament cells, decreased nitric oxide expression, and decreased pro-inflammatory cytokine production in human periodontal ligament cells induced by *P. gingivalis*(68). The antibacterial properties of blackberry extract show a reduction of metabolic activity in *P. gingivalis, F. nucleatum*, and *S. mutans* (69). Thus, certain nutrients modulate the human immune system and alter the oral biofilm formed by *P. gingivalis* improving periodontal health. Molecules active in these extracts might be exploited for development of therapeutic agents.

## Role of bacteriophage and CRISPR regions on microbial community structure

Over the last few decades, the belief that bacteriophages (bacterial viruses) are key to maintaining homeostasis in microbial populations has been developing (70–72). Growing evidence suggests that viruses play a significant role in bacterial population control as well as in the transfer of DNA in oral biofilms (73–76). A large proportion of viruses recovered from the oral cavity belonged to the order of bacteriophages and contain integrases, likely indicative of viral DNA integration into bacterial genomes and replication with the bacterial host (75). Furthermore, it has been observed that viral DNA contains genes homologous to genes involved in bacterial pathogenesis, suggesting that viruses may serve as vehicles for transfer of pathogenic gene function among closely related bacteria (75–77). Hence, exchange of viral DNA as a mechanism of horizontal gene transfer within the oral cavity is potentially a key area of investigation (76). Within the biofilm there is an abundance of DNA either through active bacterial secretion, contributing to biofilm matrix structure, or passive self-induced or viral-induced cell lysis, thus the opportunity for free DNA uptake is likely occurring. In addition, the physical restraints and close proximity of bacteria in a biofilm facilitate the intra- and inter-species transfer of DNA (76). *P. gingivalis* is thought to have a plastic genome, and there is a great deal of strain-to-strain genome variability (78–80). A recent report suggested that most horizontal gene transfer in *P. gingivalis* occurs with free DNA uptake with viruses not playing a major role. However, given the latest research on viruses in the oral cavity, this will likely be challenged (81). As of yet, no bacteriophage has been identified for *P. gingivalis*, but the natural cell-lysis observed in culture remains to be classified as either autolysis or phage-mediated. At this point, what remains unknown is how viruses in the oral cavity contribute to the development of periodontal disease, as a number are known vectors of disease (82). It has been noted in other disease models that viral communities play a key role in disease development; specifically, the viral communities, as well as the bacterial, in the respiratory tract of cystic fibrosis patients differs greatly from those of healthy individuals (83–85). There is an unmet need to understand the dynamics between viruses and bacteria in the human oral cavity because viruses likely help to shape the structure of the human oral microbiome.

The characterization of the Clustered Regularly Interspaces Short Palindromic Repeat (CRISPR) regions of bacterial genomes has begun to provide insight into interactions between viruses and bacteria. Found in most bacteria and archaea, CRISPR loci, together with Cas proteins, are a bacterial defense mechanism against viruses and conjugative plasmids (86). CRISPR systems vary greatly among microbial species and repeat sequences as well as *cas* genes are very divergent from bacterium to bacterium in terms of sequence, but also in terms of spacer sizes and the number of repeats (87). The Human Microbiome Project (HMP) has enabled exploration of the diversity and distribution of CRISPRs in human-associated microbial communities. CRISPRs were detected in all human sites, and it was observed that CRISPR spacers are actively acquired in both gut and mouth (88, 89).

The evolution and occurrence of diverse CRISPRs have been an active area of research in human oral microbiome studies. CRISPRs have been identified that are unique to the oral cavity and have been so far observed in *P. gingivalis*, as well as in streptococcal spp., *Aggregatibacter actinomycetemcomitans*, *Filifactor alocis*, and oral spirochetes (74, 80, 89–92). Interestingly, although not surprising due to the known extensive genome rearrangements, the two sequenced *P. gingivalis* strains have very different CRISPR systems (80). The complicating factor related to the study of CRISPRs within the oral microbiome is that, although samples over time from the same individual and oral site share the most spacers, spacers from different individuals are not common (75, 77, 91). Hence, the evolution of CRISPRs appears to be shaped by subtle environmental changes and the life histories of bacterial populations. Essentially, CRISPRs can be thought of as a bacterial immune system to counteract oral viruses as well as a way to sustain the bacterial population. The presence of these gene loci in *P. gingivalis* provides researchers with a number of tools for population studies, specifically CRISPR information can be used to track populations over time, to infer the ecology and evolution of *P. gingivalis* within a periodontal pocket, and to examine how this contributes to the disease progression or as a reference to discover biomarkers for disease.

## Parallels between periodontal biofilms and the cellular communities that lead to cancer

Microbial biofilms coexisting with their human hosts are dynamic cellular communities that mimic tissues that evolve over one's lifetime (93–95). In cases leading to disease, this evolution reflects shifts in the bacterial population composition that result in increased pathogenesis, e.g. antibiotic resistance, alteration of host immunity, etc. Similar changes are seen with the development of cancer, another progressive, age-dependent disease that reflects an evolving community of cells that essentially becomes a tissue ‘ignored’ by host immunity. In addition to transformed cells, tumors include normal cells, parenchyma, stroma, and immune cells, whose presence are essential for cancer development. Thus, both periodontal disease and cancer reflect age-dependent changes in the composition and function of persistent cellular communities that evolve to a point of pathogenic capability that no individual member would demonstrate alone. Like cancer, periodontal biofilm development needs to be dissected in its earliest stages, particularly prior to the rampant pathogenic stage, to determine what establishes the conditions required for the emergence of disease. In the following paragraphs, we discuss parallels between periodontal biofilms and the cellular communities that lead to cancer; perhaps current knowledge of cancer development can inform periodontal disease and vice-versa.

Early study of the immune system clearly showed that it has evolved to fight acute infections. However, there is growing appreciation for the ability of the immune system to ‘tolerate’ disease and establish homeostasis (96). Furthermore, there is now evidence for the immune system actually contributing to the evolution of chronic diseases, most notably cancer. In the immunoediting hypothesis, the immune system has actually been proposed to ‘groom’ or ‘educate’ the tumor, eliminating the cells that are the most readily detected, putting selective pressure on the tumor to evolve cellular variants that resist recognition (97). This takes time, in fact in most cases a lifetime. In a similar fashion one could envision how an oral biofilm could be ‘trained’ by the immune system over many years. Particular microenvironments or niches reflect experimental subcommunities tested for their ability to persist; the quality of the immune response could differ within/among these microenvironments. Eventually a tipping point would be reached where the microbial community, collectively, has acquired the characteristics necessary to subvert immunity, such as LPS variation or complement resistance, therefore affording conditions for the emergence of *P. gingivalis* and full-blown disease.

Tumors have been described as ‘wounds that fail to heal’ reflecting a switch from a pro-inflammatory response to a ‘smoldering’ inflammation or housekeeping process that actively suppresses anti-tumor immunity (98, 99). In parallel, a hallmark of periodontitis is that the disease progresses in periodic ‘bursts’ of tissue destruction followed by periods of quiescence where partial healing will occur; hence there is a pro-inflammatory, wounding event that is essential for staging the subsequent pathology. A switch from a pro-inflammatory response to an anti-inflammatory, healing scenario has also been reported for juvenile periodontal disease (100). High, pro-inflammatory cytokine production plummets in the ‘health’ to ‘pre-bone loss’ period in these patients. The healing phase includes significant collagen deposition followed by a shift in the microflora. Subsequent collagen destruction precedes the bone degradation and loss characteristic of periodontal disease. Gingivitis, which classically precedes adult periodontal disease, provides a pro-inflammatory, wounding event essential for staging the subsequent pathology mediated by *P. gingivalis* (101). The collagen deposited following recovery from gingivitis affords a prime substrate for the collagenase and Arg- and Lys-specific gingipains produced by this oral pathogen. It is significant to note that in addition to mediating significant tissue destruction, these proteases also temper host immunity by impeding reactive oxygen species (ROS) production and blocking the clearance of apoptotic neutrophils, a classic anti-inflammatory mechanism (102–104).

As the core of an evolving tumor becomes hypoxic, angiogenesis and immune suppression follow with the catabolism of critical, limiting amino acids (Arg, Trp) fostering immune suppression (98, 105–107). Likewise, oxidative stress with significant ROS generation and amino acid depletion are noted with periodontal biofilms (108–110). Furthermore, immune ‘paralysis’, reflecting antagonistic cytokine signaling, is possible as the biofilm composition changes with aging (8, 111). Two key components of the periodontal biofilm community afford a classic example of Th1-Th2 cytokine antagonism: HagB of *P. gingivalis* triggering TLR4 (Th1 response) and BspA of *T. forsythia* ligating TLR2 (Th2 response) (112, 113). Individually these responses would polarize an immune response towards a particular T cell subset; concurrently, they could promote immune paralysis.

Although the mechanisms are clearly distinct, a hallmark of both tumors and microbial biofilms is persistence in spite of therapeutic treatments; the latter have actually been proposed to serve as models for studying the dynamics and evolution of drug resistant cells within malignant tissues (114). There also is the physical challenge of getting drugs to penetrate to the core of tumors and biofilms. Particularly in response to hypoxia, cells in both biofilms and tumors deploy efflux pumps that reduce the efficacy of the drugs that penetrate to the core of these structures (115–119). Biofilms and tumors also can harbor ‘persister’ or stem cells that are metabolically dormant and thus resist drug treatment (120, 121). The successful eradication of tumors or pathogenic biofilms is particularly difficult due to the fact that the debulking or debriding of these cellular communities usually leaves residual cells that can re-establish disease (122–124). Collectively, these challenges highlight the need to develop therapeutic strategies to break the immune tolerance that these cellular communities have established. We must appreciate that the immune system held the tumor or biofilm in ‘check’ for many years. A return to that scenario is a rational therapeutic objective. The recent success of novel immunomodulatory drugs for certain cancers is validating this concept and should prove particularly effective when combined with existing treatment protocols (125–127). Perhaps these advances in oncology, and those surely to follow, can inform future therapeutic strategies for both the prevention and the treatment of periodontal disease.

## Concluding remarks

When grown in the laboratory, *P. gingivalis* produces a number of virulence determinants that clearly define this organism as pathogenic, yet the underlying factors that result in the *in vivo* switch this organism makes from ‘biont’ to ‘pathos’ is not clear. On its own, *P. gingivalis* does not induce disease in germ free mice, yet it can transform a benign microbial community to a pathogenic one leading to inflammatory periodontal bone loss. On the other hand, *P. gingivalis* has been found to colonize the gingival epithelium in the absence of periodontal disease in an otherwise healthy mouth. Hence, the pathogenic potential of *P. gingivalis* is not solely linked to its ability to grow; its role in the development of a pathogenic biofilm appears to be dependent on both its physiological state and intercellular interactions within the microbial consortium. Throughout this review we have highlighted the interconnectedness of microbes within the oral biofilm community, where different genera will actively respond to the presence or absence of one another. Also, in order to emphasize the dynamic and complex nature of the oral biofilm, we have presented parallels between human cellular communities that lead to cancer and development of pathogenic biofilms. We also remarked on how the population of *P. gingivalis* likely evolves within the periodontal pocket over a life time. To unravel such a complex biological system, requires a greater understanding of the microbial players within the community, so investment in the development of new model organisms is warranted. Moreover, given the strong foundation of knowledge regarding the oral microbiome, research in this area will likely lead to some remarkable discoveries regarding the collective behavior of these uni-cellular organisms.
